# Implantation Failures and Miscarriages in Frozen Embryo Transfers Timed in Hormone Replacement Cycles (HRT): A Narrative Review

**DOI:** 10.3390/life11121357

**Published:** 2021-12-07

**Authors:** Dominique de Ziegler, Paul Pirtea, Jean Marc Ayoubi

**Affiliations:** Department of Obstetrics and Gynecology, Hopital Foch University of Paris Ouest (UVSQ), 92150 Paris, France; paulpirtea@gmail.com (P.P.); jm.ayoubi@hopital-foch.com (J.M.A.)

**Keywords:** frozen embryo transfers, progesterone, subcutaneous (SC), self-injected, hormone replacement

## Abstract

The recent advent of embryo vitrification and its remarkable efficacy has focused interest on the quality of hormone administration for priming frozen embryo transfers (FETs). Products available for progesterone administration have only been tested in fresh assisted reproduction technologies (ARTs) and not in FET. Recently, there have been numerous concordant reports pointing at the inefficacy of vaginal preparations at delivering sufficient progesterone levels in a sizable fraction of FET patients. The options available for coping with these shortcomings of vaginal progesterone include (i) rescue options with the addition of injectable subcutaneous (SC) progesterone at the dose of 25 mg/day administered either solely to women whose circulating progesterone is <10 ng/mL or to all in a combo option and (ii) the exclusive administration of SC progesterone at the dose of 25 mg BID. The wider use of segmented ART accompanied with FET forces hormone replacement regimens used for priming endometrial receptivity to be adjusted in order to optimize ART outcomes.

## 1. Introduction

The need for luteal supplementation has emerged as a clinical necessity practically from the inception of ovarian stimulations (OS) used in assisted reproduction technologies (ARTs). The mechanism by which OS impacts progesterone production took longer to be unveiled, however. We now understand that OS alters LH production by the anterior pituitary because of the use of GnRH analogues—agonists and antagonists—and excessively high hormonal levels induced by OS [1]. During the luteal phase, pulsatile LH production normally drives intermittent progesterone elevations occurring at a frequency of approximately one pulse every 120–180 min [2]. The absence of LH pulses as a result of OS leads to progesterone deficiency as soon as the lasting effects of hCG used for triggering ovulation wane off [3]. This, therefore, has been the ground for initiating progesterone supplementation, typically starting on the day of oocyte retrieval [1].

Over the past decade, ARTs have seen an overwhelming increase in embryo cryopreservation due to the remarkable success rates of embryo vitrification [4] and the possibility it offers to avoid ovarian hyperstimulation. This brought together new perspectives and new challenges for luteal phase support in FETs, which constitutes the core topic of the present review article. In FETs conducted using an HRT protocol, chorionic hCG cannot take over progesterone production as in fresh ART cycles because of the absence of corpus luteum. Therefore, in FETs using HRT priming, E2 and progesterone need to be effectively continued until the luteo-placental shift, which occurs at approximately10 weeks of pregnancy. Furthermore, in the case of progesterone, delivery rates have to match the increase in production seen during the early weeks of pregnancy under the control of chorionic hCG in fresh ART cycles. Conspicuously, however, none of the progesterone preparations currently approved for use in ARTs have been formally tested in FET protocols. Do the preparations tested in fresh ART cycles suffice for matching the rising progesterone levels encountered in the early weeks of pregnancy, or is there a need to increase the doses prescribed? This question is becoming particularly important in light of a flurry of recent publications pointing at the insufficient progesterone levels obtained in a sizable fraction of women undergoing FET with vaginal progesterone.

**Material and methods**: The literature was reviewed for articles addressing FETs timed in HRT, excluding transfers in natural or modified natural cycles and priming approaches not based on natural progesterone administration. Moreover, the use of synthetic progestins such as dihydroprogesterone which do not sustain full predecidual changes and are not approved in FETs, are not addressed in this review.

## 2. Fresh and Frozen Embryo Transfers (FETs)

### 2.1. Fresh Embryo Transfers

**Figure 1:**
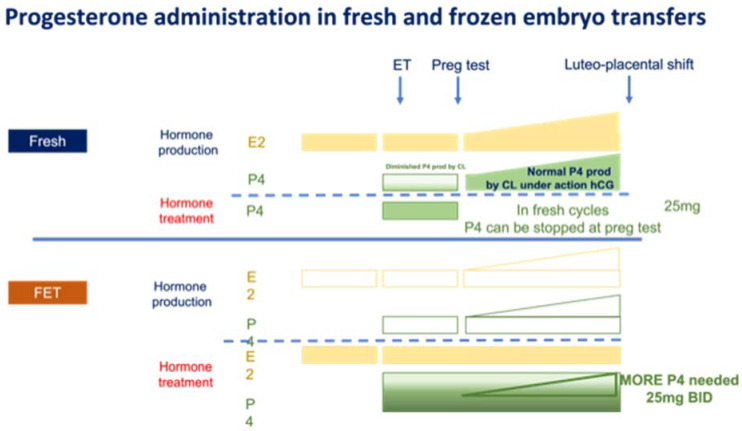
Progesterone administration in fresh and frozen embryo transfers. Hormone production and replacement in the case of fresh and frozen embryo transfers. In fresh ART, progesterone production is impaired during the luteal phase only. In FETs timed in HRT, there is no endogenous hormone production and treatment should provide sufficient progesterone to reach efficient levels.

In fresh transfer ART, the only impact of OS on progesterone production occurs during the luteal phase when pituitary LH is suppressed and fails to properly sustain the corpus luteum activity. Once pregnancy is established, however, corpus luteum activity is under the control of hCG produced by the developing embryo and is not dependent on pituitary LH anymore. Progesterone production in early pregnancy is therefore normal in fresh transfer ART. Several investigators have documented that progesterone supplementation can be stopped on the day of the positive pregnancy test [5,6] without risking an increase in the rate of pregnancy losses. Most groups, however, continue progesterone treatment for further weeks—often 10 weeks, but without good reason. Figure 1 illustrates the fact that progesterone supplementation in fresh embryo transfer ART is meant to complement the partial production deficit during the luteal phase of OS, while the production of progesterone in early pregnancy is normal.

E2 supplementation during the luteal phase of fresh embryo transfer ART is generally considered as non-necessary [7,8]. Zhao et al., however, contend that E2 supplementation may have beneficial effects in women whose E2 levels on the day of hCG trigger are <5000 pmol/L [9]. This issue of E2 supplementation in women with moderate ovarian response to OS should be pursued as it has retained little interest so far. The literature should be followed on this topic.

### 2.2. Frozen Embryo Transfers (FETs)

In FETs conducted in HRT, the situation that prevails is drastically different from that encountered in fresh embryo transfer ART. Encouraged by the outstanding results obtained in donor oocyte ART, E2 and progesterone substitution cycles have been proposed for timing FETs, particularly in oligo-anovulators. for over three decades [10]. This approach, which originally included prior ovarian suppression using a GnRH agonist in women whose ovarian function is active, was later simplified. Indeed, it was demonstrated that E2 alone suffices to prevent follicular recruitment by its antigonadotropin effects, particularly on FSH [11,12]. In light of this simplification, E2 and progesterone substitution cycles have been preferred by many ART centers for their simplicity of programming and efficacy [13]. E2 and progesterone cycles indeed allow an advanced choice of the day of transfer and a single blood serum progesterone and echographic control, also performed on a preset date. Of course, other options also exist for timing FETs, either in the natural cycle for regular ovulators or following light ovarian stimulation. Generally, these latter two approaches require more frequent controls that cannot be precisely planned in advance and are thus seen as more cumbersome.

In FETs using E2 and progesterone substitution cycles, there is no corpus luteum and therefore the treatment is the sole supplier of E2 and progesterone, as is also illustrated in Figure 1. E2 is generally provided in largely sufficient amounts in these supplementation cycles. Furthermore, our prior work challenged the true necessity for E2 presence during the luteal phase and early pregnancy [14], a fact that was later confirmed by others [15]. Recent data revealed that the situation is drastically different for progesterone, however. Indeed, vaginal progesterone which induces the full predecidual transformation of an estrogenized endometrium [11,16], has recently been shown to encounter some shortcomings in FETs, as discussed below. These observations concerning the limits of vaginal progesterone in FETs caught people by surprise and deserve further discussion in the following section of this article. Of note, all the progesterone preparations available for luteal phase support have been formally tested and approved solely in fresh embryo transfers and none in FETs—at least in the US.

## 3. Shortcomings of Vaginal Progesterone in FETs

### 3.1. Vaginal Progesterone Seen as an Alternative to Intramuscular (IM) Injections

In the first substitution cycles designed for donor egg ART, progesterone was administered by IM injections of oil-base solutions [17]. However effective, IM injections are painful and generally cannot be self-administered. The IM route originally appeared as the only one possible because of the hydrophobic characteristics of progesterone that render aqueous preparations seemingly impossible. Therefore, alternatives to IM progesterone have been looked for. Oral progesterone is not a viable option because while nearly 100% is absorbed when orally absorbed in a micronized form, it is nearly entirely metabolized during the first liver pass. Of note, the important hepatic metabolism during the first liver pass is not always evident because of erroneous blood measurements due to the high levels of progesterone metabolites [18]. Likewise, transdermal progesterone cannot replace IM injections. Indeed, the daily production of progesterone—25 mg/24 h in the midluteal phase—exceeds by two orders of magnitude that of E2—0.05 to 0.5 mg/24 h—making the manufacturing of transdermal patches impossible. Hence, vaginal administration appeared as the sole viable alternative to the painful IM injections and its use has therefore been pursued for practicality’s sake.

### 3.2. First Uterine Pass Effect

Exploring the vaginal administration of progesterone in order to define the characteristics of this alternative to the painful injections of IM progesterone in oil preparations has led to unexpected findings. Miles et al. were the first to report that the uterine concentration of progesterone far exceeded expectations in the case of vaginal administration [19]. In their report, plasma progesterone levels were higher in the case of IM administration, but uterine concentration were more elevated in the case of vaginal delivery. The authors postulated that progesterone might be directly transported form the vagina to the uterus, a phenomenon now referred to as the first uterine pass effect. As uterine concentrations were calculated on samples obtained by transvaginal uterine aspiration, some argued that the biopsies might have been contaminated by progesterone present in the vagina. To clarify this issue, a similar experiment was repeated but this time by obtaining uterine tissue at the time of hysterectomy, therefore excluding possible contamination by progesterone present in the vagina [20]. An ex vivo study using labeled progesterone indeed verified that progesterone effectively traveled from the vaginal cuff to the uterine corpus [21]. Based on these and other studies, we proposed that the direct vagina-to-uterus transport occurred by a countercurrent exchange mechanism of the type existing, for example, in Henle’s loop of the kidney [22].

Practically, unveiling the first uterine pass effect of vaginally administered progesterone quelled—but for a time only—the concerns about the relatively low circulating levels of progesterone achieved by vaginal administration. As discussed below in further detail, serum progesterone levels are also important for the development and maintenance of pregnancy in FETs.

### 3.3. Shortcomings of Vaginal Progesterone in FETs

Devine et al. were first to point at the shortcomings of vaginal progesterone in FETs using a prospective randomized trial [23,24]. These authors tested the vaginal preparation Endometrin^®^ (Ferring Pharmaceuticals, San Diego, CA, USA) at the approved dose of 100 mg BID recommended for fresh embryo transfers in a prospective randomized trial. The comparators were (i) IM progesterone (50 mg/day in oil) and (ii) a ‘combo’ regimen using vaginal progesterone as above together with IM progesterone (50 mg) on the day of the embryo transfer and every third day thereafter. A power calculation led to recruiting 1170 subjects who were randomized between the three arms in a 1:1:1 ratio. An interim analysis planned after 50% recruiting revealed that clinical pregnancy rates and overall pregnancy losses were statistically lower and higher, respectively, in the group receiving vagina progesterone only [23]. The vaginal progesterone only arm was thus stopped. Completion of the study confirmed that there were no differences between the group receiving IM progesterone or the ‘combo’ preparation at the study’s end [24].

At approximately the same time, Labarta et al. reported that a fraction of women receiving vaginal progesterone—400 mg BID—had serum progesterone <9.2 ng/mL on the day of the transfer [25]. These women, whose serum progesterone was lower, also had lower pregnancy rates and higher miscarriage rates [25]. These findings of lower ART outcomes in women whose serum progesterone was lower than 10 ng/mL were later confirmed by others [26].

Increasing the dose of vaginal progesterone is not an option for elevating serum levels. Indeed, Archer et al. reported that the administration of progesterone from 100 mg or 200 mg suppositories achieved similar serum levels [27]. These authors conclude that the vaginal mucosa or the total surface area of the vagina is the limiting factor for the absorption of progesterone from the vagina. Likewise, we demonstrated that differences in concentration of the progesterone preparation induced differences in serum concentration, but not differences in the amount of the same concentration preparation [28]. Taken together, these results indicate that it is the concentration of the progesterone preparation used that drives absorption, not the amount of product used. Increasing the number of daily administrations of vaginal progesterone may, however, increase serum concentrations [29].

### 3.4. Pelvic and Non-Pelvic Effects of Progesterone

The effects of serum progesterone levels on pregnancy and miscarriage rates in FETs are intriguing considering that the uterine concentration of progesterone is high as a result of the first uterine pass effect [20,22]. This discordance between the high uterine concentration of progesterone and the dependance of pregnancy and miscarriage rates on serum levels of progesterone is therefore truly intriguing.

**Figure 2:**
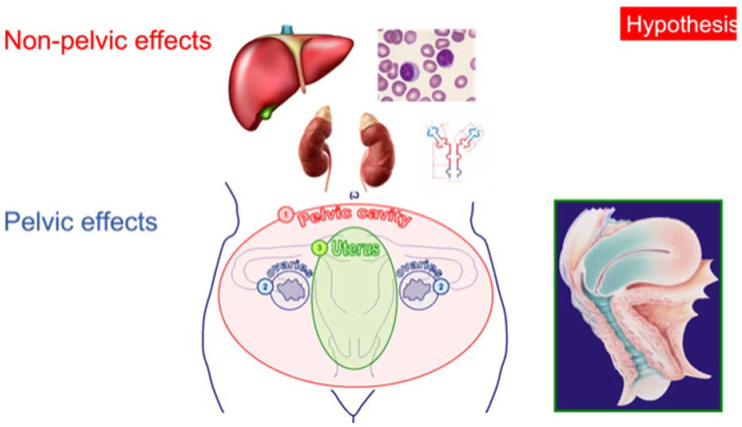
Pelvic and non-pelvic effects of progesterone. A hypothesis is proposed to explain that low circulating levels encountered with vaginal progesterone administration are counterproductive despite high uterine tissue concentration. We propose that immune modulation effects of progesterone—necessary for pregnancy development—are dependent upon circulating levels, as it is mediated outside of the pelvic area.

This discordance between the impact of uterine concentration of progesterone (high) and serum levels (variable) on FET outcome led us to formulate a new provocative hypothesis. Until now, we probably erroneously believed that solely the pelvic concentration of progesterone counted for embryo implantation and pregnancy development. This was ignoring the role played by progesterone in generating the state of immune tolerance necessary for pregnancy development. The latter depends not only on the pelvic [30] but also on the non-pelvic actions of progesterone [31]. Developing the state of immune tolerance that characterizes pregnancy implies effects of progesterone that are exerted on the bone marrow, liver, and adrenals, which all depend on serum levels of progesterone, not uterine concentration [31]. It is therefore possible, if not likely, that we have all along been oblivious of these non-pelvic effects of progesterone, only concentrating on the pelvic effects. These views on the roles of the pelvic and non-pelvic effects of progesterone are illustrated in Figure 2. The recent data on the impact of progesterone levels <10 ng/mL on pregnancy and miscarriage rates speak to the clinical relevance of serum progesterone on the non-pelvic effects of progesterone.

## 4. A New Subcutaneous (SC) Progesterone Preparation

**Figure 3:**
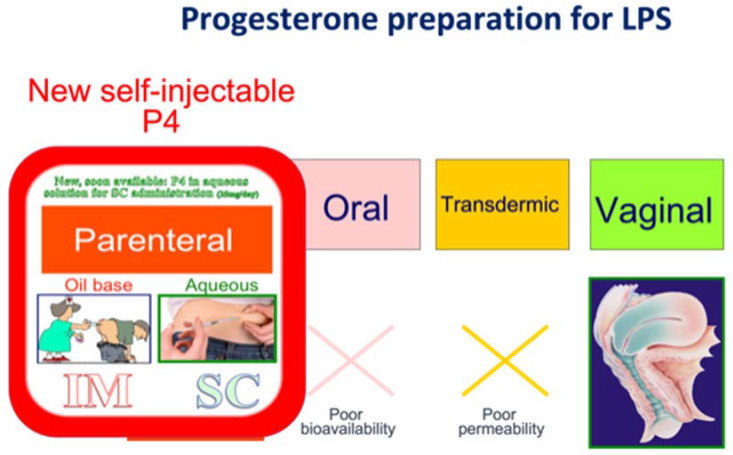
Progesterone preparation for Luteal phase support. Options available for progesterone administration today. New subcutaneous administration of progesterone in aqueous solution is an alternative to IM injection of progesterone in oil-base solutions. The dose of Prolutex^®^ 25 mg BID is equivalent to IM injection of 50 mg daily. Alternatively, progesterone can be administered vaginally with daily rescue with Prolutex^®^ 25 mg.

Despite being seemingly impossible due to the highly hydrophobic characteristics of progesterone, an aqueous preparation has recently been proposed [32]. The preparation of the aqueous solution was made possible by encapsulating progesterone molecules in a starch residue product—cyclodextrin—which confers the necessary polarity for a hydro soluble preparation. Following injection, the cyclodextrin moiety is readily digested, which liberates native progesterone in the blood stream. The developed product—Prolutex^®^ (IBSA Switzerland)—being in aqueous form is thus easily self-administered by SC injection (Figure 3).

Pharmacokinetic data on Prolutex^®^ 25 mg administered subcutaneously daily for 11 days indicate that the nadir levels of progesterone are at approximately 6 ng/mL. Clinical trials have validated that the daily dose of 25 mg of SC progesterone induces the full predecidual transformation of an endometrium primed with E2 only [16]. These findings therefore confirm that Prolutex^®^ administered at the dose of 25 mg/day is adequate for priming endometrial receptivity. These data, however, fail to inform as to whether the dose of 25 mg/day suffices for coping with the rising progesterone levels in early pregnancy. Confirming this view, the dose of 25 mg/day was proven effective in fresh-transfer ART in two large phase III non-inferiority trials against approved vaginal progesterone preparations [33,34]. At this dose—25 mg/day—the product has not been formally tested in FETs

## 5. Rescue Options

The numerous concordant reports on the shortcomings of vaginal progesterone used for luteal phase support in FETs led investigators to look for corrective measures. In a prospective trial, Alvarez et al. undertook supplementing women whose progesterone was <10.6 ng/mL on the day prior to embryo transfer with subcutaneous injections of progesterone (Prolutex^®^ 25 mg/day, IBSA Switzerland) [35]. Embryo transfer was performed on the following day after verifying that progesterone levels were >10.6 ng/mL following the subcutaneous injection of progesterone. Women whose progesterone levels were >10.6 ng/mL on the day prior to embryo transfer continued their hormone replacement using the sole administration of vaginal progesterone [35]. Ultimately, there were no differences in live birth rates (LBRs) between women whose low progesterone levels were rescued with SC progesterone (25 mg/day) and those whose progesterone was >10.6 ng/mL [35]. These data indicate that subcutaneous progesterone rescue in women whose progesterone serum progesterone levels are too low—practically, <10 ng/mL—is effective at correcting pregnancy and miscarriage rates. Similar favorable results following rescue with subcutaneous progesterone were reported by Yarali et al. [36]. These authors reported diminished ART outcomes in women whose serum progesterone was <8.75 ng/mL on the 5th day of vaginal progesterone administration [36]. In this case-control study, 40 women with low serum progesterone received additional daily sub cutaneous (SC) administration of 25 mg of progesterone (Prolutex^®^, IBSA, Switzerland), starting on the 5th day of progesterone administration. Mean progesterone level on the 6th day of progesterone administration was raised to 33.43 ng/mL (range of 14.6–82.6) [37]. The results observed following rescue with SC progesterone were compared to those of age- and BMI-matched controls (3 to 1) with progesterone levels >8.75 ng/mL on the 5th day of vaginal progesterone administration. The comparison showed no difference in either pregnancy or miscarriage rates [37]. Finally, the original authors—Labarta’s group in Valencia, Spain, who were the first to blow the whistle on the role of serum progesterone levels in FETs—confirmed that rescue options are effective [38].

## 6. Subcutaneous Progesterone for FETs

Turgut et al. reported a retrospective, single-center cohort study, comparing pregnancy outcomes of 507 AC-FET cycles performed using either IM (50 mg/day) or SC progesterone 25 mg BID (Prolutex^®^) [39]. The study was conducted between June 2018 and April 2020 in a group who had switched to a systematic freezing of all embryos and deferred embryo transfer policy. All participant women were <37 years of age, excluding patients with repeated miscarriages. The choice between IM and SC progesterone was made according to doctors’ preferences (not randomized). There were no differences between the IM and SC groups in positive pregnancy rates (*p* = 0.474), clinical pregnancy rates (*p* = 0.979), LBRs (*p* = 0.404), and miscarriage rates (*p* = 0.144). LBRs were 58.7% and 62.7% in women receiving IM and SC progesterone, respectively [39].

The findings of this study [39] are important for all practitioners who exclusively use injectable progesterone for optimal results. The advent of SC progesterone, therefore, offers, in light of the results reported by Turgut et al., a true alternative to the painful IM injections [39]. The major limitation of this study is its retrospective design, however. One should therefore await the results of currently ongoing prospective trials comparing IM and SC (Prolutex^®^) progesterone for luteal phase support in FET ARTs.

## 7. Conclusions

Existing progesterone preparation products have all been formally tested and approved for luteal phase support in fresh ART cycles only. By extrapolation, these products have, however, been used in FET cycles. In fresh ARTs, many groups—the vast majority in certain European countries—have opted for using, nearly exclusively, vaginal progesterone for avoiding the painful IM injections. The rapid extension of FETs due to the improvement of cryopreservation techniques has led to unveiling the shortcomings of vaginal progesterone when used for priming receptivity in FET cycles conducted in the HRT model. Indeed, unexpectedly recent data revealed that progesterone level requirements for progesterone administration in FETs are higher than those obtained in large fractions of women receiving vaginal progesterone only. In 25–33% of women, depending on the study, higher progesterone levels are necessary for optimizing FET cycle outcomes, maximizing LBR, and minimizing early pregnancy losses. This is probably due to the fact that the non-pelvic effects of progesterone—for inducing pregnancy-specific immune tolerance—are dependent upon sufficient plasma progesterone levels.

Two options have been proposed for coping with the shortcomings of vaginal progesterone in FET: first, a rescue option has been proposed with the addition of SC progesterone (Prolutex^®^) 25 mg/day if progesterone levels are <10 ng/mL. In order to avoid the need for measuring progesterone levels on the 5th day of progesterone administration, some have opted for a systematic combo—vaginal and SC progesterone—option [40]. Second, Turgut et al. demonstrated that IM progesterone can be replaced by twice daily administration of SC progesterone (Prolutex^®^) [39].

Taken together, these data indicate that vaginal progesterone is not adequate for luteal phase support in FETs and options adding injectable progesterone (notably, SC progesterone) are necessary. Further, possibly multicentric, studies are awaited to clarify this issue.
